# Quantitative and Qualitative Changes in the Deformed Wing Virus Population in Honey Bees Associated with the Introduction or Removal of *Varroa destructor*

**DOI:** 10.3390/v14081597

**Published:** 2022-07-22

**Authors:** Luke Woodford, Craig R. Christie, Ewan M. Campbell, Giles E. Budge, Alan S. Bowman, David J. Evans

**Affiliations:** 1 Biomedical Sciences Research Complex, University of St. Andrews, St. Andrews KY16 9ST, UK; d.j.evans@st-andrews.ac.uk; 2 Institute of Biological and Environmental Sciences, School of Biological Sciences, University of Aberdeen, Aberdeen AB24 3FX, UK; craig.christie@abdn.ac.uk (C.R.C.); e.m.campbell@abdn.ac.uk (E.M.C.); a.bowman@abdn.ac.uk (A.S.B.); 3 School of Natural and Environmental Sciences, Newcastle University, Newcastle NE1 7RU, UK; giles.budge@newcastle.ac.uk

**Keywords:** honeybee, deformed wing virus, *Varroa destructor*, miticide, virus diversity, colony management, sequencing

## Abstract

*Varroa destructor* is an ectoparasitic mite associated with significant losses of honeybee colonies globally. The mite vectors a range of pathogenic viruses, the most important of which is the Deformed wing virus (DWV). In the absence of *Varroa*, DWV exists as a low-level, highly diverse virus population. However, when transmitted by *Varroa,* certain variants become highly elevated, and may become near-clonal and cause symptomatic infections. Mite transmission between colonies can occur when parasitised workers drift from or rob adjacent hives. These activities can result in elevated mite levels, but the resulting change in the DWV population, the primary determinant of winter colony losses, has not been determined. In reciprocal studies, we investigated the influence of the removal of mites, or their acquisition, on the DWV population. When mites were removed from heavily infested colonies, there was a striking and rapid reduction in virus load. Conversely, siting *Varroa*-naïve colonies in a mite-infested apiary resulted in the acquisition of mites and concomitant changes in the virus population. We observed both near-clonal and highly divergent virus populations regardless of titre, suggesting changes were stochastic and colony-specific. Our findings have implications for the outcome of strategies in areas with total or patchy implementation of *Varroa* control plans.

## 1. Introduction

The ectoparasitic mite *Varroa destructor* was originally a parasite of the Asian honeybee (*Apis cerana*), before jumping hosts to infest European honeybee (*Apis mellifera*) colonies about a century ago. Since then, *Varroa* has become near-globally distributed with the movement of managed hives [1]. The mite is a vector for a range of honeybee pathogens, most notably Deformed wing virus (DWV) [2]. DWV is an endemic positive-sense single-stranded RNA virus observed at low viral titres of <10^6^ genome copies/bee, levels which rarely, if ever, cause symptomatic disease [3,4]. However, the presence of *Varroa* drastically alters the dynamics of infection [5,6]. The virus is transmitted from mite to host when *Varroa* feed on developing pupae, causing a dramatic increase in viral load and symptomatic infections (titres can reach 10^13^ copies, and symptoms are typically observed when DWV > 5 × 10^6^ genome copies/bee [7]), and increased mortality [8,9,10]. Colonies with high levels of *Varroa* infestation, and associated elevated DWV levels, are the major cause of overwintering colony losses due to the reduced longevity of the winter bees [11].

Controlling *Varroa* infestations significantly reduces the incidence of DWV-mediated pathogenesis and markedly reduces colony losses. However, the remaining *Varroa* reproduce, necessitating annual, or more frequent, mite management. In addition, mite levels may be further amplified through the acquisition of ‘phoretic’ mites from workers drifting during orientation flights, or by the activities of foragers robbing nearby collapsing (mite-infested) colonies [12,13]. An analysis of marked workers revealed that as many as 42% of workers in a colony could be alien due to drifting [14], and this is exacerbated when colonies are kept in close proximity [15]. Acceptance of drifting workers is further exacerbated by a high mite reinfestation rate, with rates of ~76 mites a day recorded in mite-free colonies over 200 m away from the nearest infested hives [16]. Additionally, colonies with high *Varroa* infestation show an increased acceptance of drifting workers, allowing uptake of other associated pests and pathogens to occur [17].

Effective miticide treatments exist, and the use of tau-fluvalinate strips to treat mite-infested colonies with high DWV resulted in a 1000-fold virus reduction compared with neighbouring, untreated colonies [18]. However, DWV levels increased again as mites re-infested the colonies throughout the active bee season.

In addition to significant increases in viral load, *Varroa* infestations have been reported to be associated with a reduction in DWV diversity [5,6]. Low-level, diverse DWV populations are superseded by high titre near-clonal virus populations when *Varroa* alter the virus transmission route, something which can be replicated by injection [6]. More recently, high DWV diversity has been observed in infected pupae with high viral titres [19,20], suggesting that the shift in virus diversity in the presence of *Varroa* is not a binary event and that other factors can influence the DWV population dynamic [21].

DWV exists as two frequently reported variants, DWV Type-A [3] and DWV Type-B (sometimes referred to as VDV-1) [22], which can seemingly freely recombine and exhibit extensive genetic similarity (>80% and >90% at the nucleotide and protein level respectively) resulting in a variable and dynamic virus population [6,21,23,24]. Recent research suggests both DWV types are equally pathogenic and cause similar wing deformities in developing bees [7,25]. Various studies have suggested that Type B is an emerging variant that is starting to dominate in honeybees in the USA and Europe, co-existing with or replacing the Type A variants [26,27,28]. One factor that may influence mite-mediated transmission is whether the viruses can replicate in the vector *Varroa*. Using an engineered recombinant DWV, Gusachenko et al. (2020) demonstrated reporter gene expression, and hence, virus replication in mites fed virus using an artificial feed packet system. Other studies have suggested that DWV Type-A does not replicate in mites and is instead vectored in a non-propagative manner [29].

It has been shown that symptomatic DWV infections, which cause over-wintering colony losses, can persist after mite removal. Additionally, it was found that low mite levels may not translate to low DWV levels, meaning some colonies could still collapse over winter after mite removal [30]. To investigate this, we combined a hive management technique (a mid-season ‘shook swarm’) with standard miticide treatments to remove *Varroa* and measured subsequent changes in the DWV population in the colonies to determine improvements in colony health. A ‘shook swarm’ is a standard beekeeping technique that separates the adult workers and the queen from the brood, comb, and stores. It is used to encourage the colony to draw fresh comb and may help eliminate brood diseases such as European foulbrood [31]. We reasoned that, since 90% of mites are typically associated with developing pupae in capped cells [32], a shook swarm, and simultaneous miticide treatment would precipitously reduce the mite population in the colony. We were interested in determining the consequences of this on the residual DWV population.

We show that this treatment combination significantly reduces *Varroa* levels in treated colonies. More importantly, within one brood cycle, the residual DWV level was reduced to a very low level and remained low for the remainder of the season. In reciprocal studies, we demonstrated that healthy *Varroa*-naïve colonies become rapidly infested when placed near mite-infested colonies, resulting in changes to the virus population and high colony losses. We observed mixed populations of high-titre DWV infections in symptomatically infected workers, indicating that not all symptomatic infections are caused by clonal viral populations. Despite differences in DWV levels between seasons, the colonies invariably died after a period of high DWV infections and no *Varroa* treatment interventions. These studies demonstrate a novel method of significantly reducing mite and virus loads rapidly to improve colony health. In parallel, we demonstrate the speed with which *Varroa* are acquired and how their introduction alters DWV levels in infested colonies. These studies highlight the importance of using timely and effective interventions to improve or maintain honeybee colony health and can be implemented using standard beekeeping methods.

## 2. Materials and Methods

### 2.1. Shook Swarm Method and Miticide Treatments

The emerging brood was sampled from each of ~12 colonies and analysed by qPCR to determine which colonies in the infested apiary had the highest DWV levels and, therefore, presumably high mite levels. Those with the most consistently elevated DWV yields were selected for the experiment (data not shown).

The shook swarm process was conducted after locating and caging the queen for safety. The hive was moved to an adjacent stand and replaced with a brood box containing frames with foundation and two amitraz-impregnated Apivar strips (Véto-Pharma, Palaiseau, France). All bees from the original hive were shaken into the new brood box, which was sealed with the caged queen, and moved to a quarantine apiary approximately 130 km away. The queen was released the following day, and the colony fed one gallon of thin syrup to encourage the drawing of new comb. Apivar strips were removed after six weeks. The apiary housing the newly positioned colonies had very low mite levels and was monitored throughout the study. Mite counts were performed by recording the number of dead mites observed on the floor of each colony.

### 2.2. Healthy’ Colonies Placed in Varroa Infested Apiary

Each year, four colonies were treated with an approved miticide in the University of St Andrews apiary in 2017 and 2019. The dead mites were counted for each hive after the treatments to establish infestation levels. A worker brood sample was taken to analyse the virus levels for each colony before they were transferred to the mite-infested apiary. The colonies were placed in the centre of the apiary, which contained between 15–18 colonies each year. No colonies in the infested apiary received any miticide treatments, although drone trapping was occasionally used to gather mites. Emerging worker brood was sampled approximately every brood cycle until the queens had stopped laying eggs for winter (July, August, September, and October).

### 2.3. Tissue Storage, RNA Extractions and cDNA Synthesis

All emerging brood samples were snap-frozen and stored at −80 °C. For the Shook Swarm experiment, dead *Varroa* were collected in 50 mL falcon tubes 24 h after the first treatment and frozen at −80 °C. Frozen samples were homogenized using the Precellys Evolution tissue homogenizer (Stretton Scientific Ltd., Alfreton, UK) for 3 × 15 s at 10,000 rpm with 20 s pauses between each pulse. RNA extractions from whole individual bees were performed using the GeneJET RNA purification kit (Thermo Fisher, Waltham, MA, USA) as per the manufacturer’s instructions. Total RNA from individual workers was quantified and quality assessed based on spectra cleanliness and a 260/230 ratio using the Nanodrop-1000 and stored at −80 °C. Total cDNA was synthesized from 1μg RNA per sample using the qScript cDNA synthesis kit (QuantaBio, Beverly, MA, USA) following the manufacturer’s protocol using oligo(dT) and random primers.

### 2.4. qPCR and PCR Amplification of DWV

In measuring absolute viral load, quantitative polymerase chain reactions (qPCRs) were performed for DWV in a Bio-Rad CFX96 Touch Real-Time PCR Detection System (Bio-Rad, Hercules, CA, USA). Reactions consisted of 1 × Luna Universal qRT-PCR master mix (New England Biolabs, Ipswich, MA, USA), 0.25 µM forward and reverse primers, and 100 ng of cDNA in a final volume of 20 µL. The DWV forward primer was 5′-ATATAGGTTCGGCTGGATCTCC-3′, and the reverse primer was 5′-TTCCAGATGCACCACACATGC-3′, amplifying a region of 150 bp in the helicase. Amplification was performed using the following thermal profile: 1 min at 95 °C, followed by 40 cycles of 15 s at 95 °C, and 30 s at 60 °C. Post amplification melting curve analysis was used to check for non-specific amplification (60–95 °C increasing 0.5 °C every 5 s). Negative template controls and a serial dilution of a positive control standard were included in each run. DWV genome equivalents were calculated from the standard curve generated from a serial dilution of a cDNA clone control obtained from 1µg of DWV VVD RNA transcript as per [7], with a linear range of 10^10^–10^3^ GE/μg.

DWV and actin end-point PCR assays were carried out in 25 µL reactions containing 10 × PCR buffer, 0.2 mM dNTPs, 0.4 µM forward and reverse primer, 2U Taq DNA polymerase (New England Biolabs, Ipswich, MA, USA) and ~100 ng of cDNA template. The DWV forward primer was 5′-CAGTAGCTTGGGCGATTGTTTCG-3′, and the reverse primer was 5′-CGCACTTAACACACGCAAATTATC-3′, amplifying a region of 1510 bp in the helicase. The actin PCR assay was performed as per [33]. An initial denaturation step of 94 °C for 20 s was followed by 30 cycles of 94 °C for 15 s, 57 °C for 20 s, and 68 °C for 2 min. This was followed by a final extension at 68 °C for 10 min. Samples were visualised using 1% agarose gel electrophoresis. Long-amp amplification for full genome fragments was carried out as per [33]. Phylogenetic analysis of sequenced amplicons was performed using Geneious Prime 2019.1.3.

### 2.5. ShoRAH Analysis of Viral Diversity and SNV Calling

Large DWV Amplicons (>10 KB) were purified, and each sample was barcoded (NCBI BioProject: PRJNA811438). Illumina Hi-seq (2 × 300 bp) paired-end reads were processed from these amplicons at the University of St Andrews. Illumina reads were then extracted and trimmed using Geneious Prime (v.2019.1.3) and extracted as a single fasta file for each honeybee sample. Haplotype diversity was calculated using ShoRAH (Short read assembly into haplotypes) with all settings run as default [34] and all haplotypes comprising >3% of the viral population included in the final population diversity analysis. This threshold was determined using a positive control cDNA clone sample which produced a 97% haplotype match.

Single nucleotide variants (SNVs) were called using statistical modelling at a frequency lower than the error rate of sequencing. ShoRAH analysis includes a statistical test of strand bias by utilising a Fisher’s exact test and a Benjamini-Hochberg correction process, which rejects any *p*-values < 0.05 in the final analysis of SNVs. Based on this method, combined with the probabilistic clustering method implemented by ShoRAH, SNVs can be called from deep sequenced viral populations with high accuracy [35]. We utilized this method to evaluate the sequence diversity changes observed in colonies over time.

### 2.6. Statistical Analysis of DWV Changes

All models were implemented using the *nlme* package in R [36,37,38]. We used linear and mixed effect models to investigate the changes in log DWV levels in colonies that had either received a shook swarm treatment or been placed in a *Varroa*-infested apiary. First, we used a linear model with the DWV level as a response variable and sampling month as the independent variable. Second, we created a mixed effect model by extending the linear model with the colony as a random effect. Model fit was compared using the Akaike Information Criterion (AIC) and 95% confidence intervals of the estimates calculated using the *nlme: intervals* function.

## 3. Results

### 3.1. Consequences for the DWV Viral Load of Rapid and Effective Varroa Removal Using a Combined Shook Swarm and Miticide Treatment

In May 2018 and 2019, we conducted shook swarms on a total of six colonies of equal strength (based on the number of brood frames and colony size) and mite burden (three in each season) from a mite-infested apiary in which *Varroa* control was not used. The queen was caged, and workers and nurse bees were shaken into a new hive with foundation only, containing two strips of amitraz-containing miticide. The queen was returned to the colony, which was relocated to a quarantine apiary and fed ad libidum with sugar syrup to encourage the production of a fresh comb.

**Figure 1:**
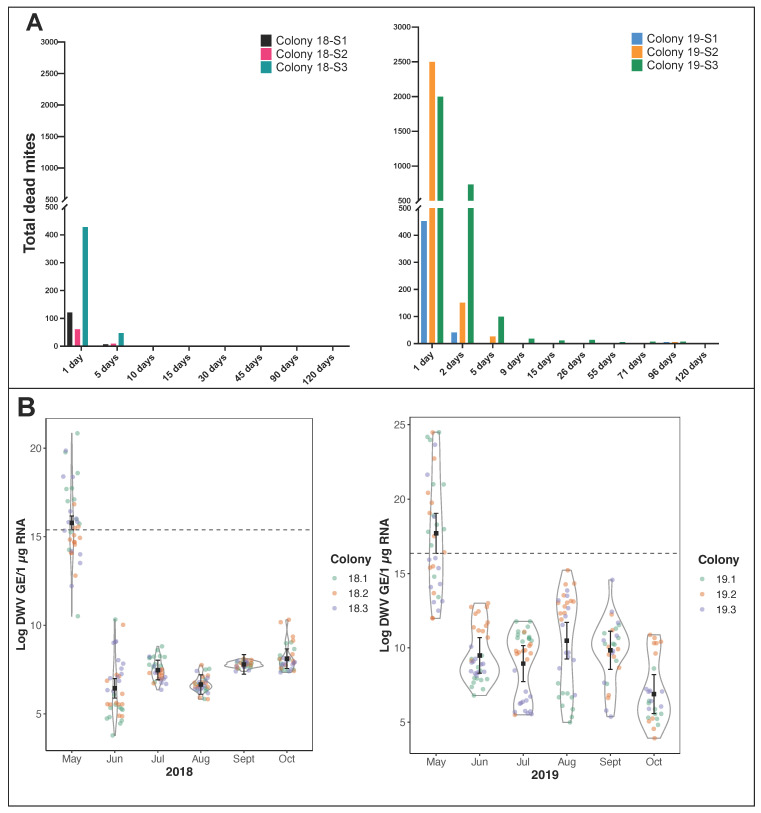
Varroa drop post-treatment and log DWV levels in emerging brood before and after shook swarm and Apivar treatments. The shook swarm and Apivar treatments were applied to the colonies after the first timepoint. (**A**). Varroa drop was counted every few days in 2018 (left) and 2019 (right) after the shook swarm and Apivar application. Additional time points where no Varroa was observed omitted for simplicity. (**B**). A violin plot showing the log DWV load in workers as measured each month after treatment. Coloured circles represent individual honeybee samples from each colony (*n* = 12). The black square and line represent the predicted mean from the mixed-effects model for each time point with corresponding 95% confidence intervals around the mean. Estimates and 95% confidence intervals below the dashed line indicate they are below the lower 95% confidence interval from the pre-treatment.

The mite drop was recorded 24 h post-treatment and periodically over subsequent weeks until the colony stopped rearing brood in October. In both seasons, the mite drop immediately after treatment was high but rapidly decreased and then remained low for the remainder of the season (Figure 1A). The mite levels in 2019 were up to 25 times higher than the preceding year, and if it is assumed that only ~10% of the mites are phoretic in a brood-rearing colony, it would indicate a total mite load in 2019 of up to ~30,000 in colonies #19-S2 and #19-S3.

Prior to conducting the shook swarm experiments, a screen of colony DWV levels was carried out using qRT-PCR analysis of 12 emerging brood samples per colony in the infested apiary. The DWV copy numbers were broadly reflective of the *Varroa* load in the screened colonies, being higher in 2019 than in 2018, with an average virus level of >10^6^ genome equivalents (GE)/μg RNA observed in all colonies. In 2018 the DWV level ranged from 7 × 10^5^–1 × 10^9^ GE/μg RNA across the three colonies, but in 2019 the range was 2 × 10^5^–4 × 10^10^ GE/μg RNA. Post-treatment, no emerging brood showed DWV disease symptoms, and average DWV levels in the samples analysed were all <10^6^ GE/μg RNA for the remainder of the season during which brood was present. 

Both the simple linear models and the mixed models indicated a significant decrease in the log levels of DWV in sampled bees over time in 2018 and 2019 (Tables S1 and S2, Figure 1B). The lowest AIC varied between models, with the simple linear model being lower than the mixed effect model in 2018 (lm AIC = 695; GLMM AIC = 706), but the reverse being true in 2019 (lm AIC = 936; GLMM AIC = 924). This indicated that the simpler lm was a better explanation of the data in 2018 but the random effect of the colony was useful to account for the effect of the colony in 2019. The proportion of residual variation explained by the inclusion of the random effect for colony varied from 0.05% in 2018 to 26% in 2019 (Tables S1 and S2). The estimates for mean log DWV dropped below the lower 95% confidence interval for every month post-treatment (Figure 1B), indicating the effect of the shook swarm treatment had a substantial and lasting impact on the log levels of DWV found in sampled bees.

### 3.2. Changes in the DWV Population Diversity following Rapid Varroa Removal Using a Combined Shook Swarm and Miticide Treatment

Honeybees with low DWV levels have previously been shown to have divergent populations of DWV [5,6]. To determine if there were changes in the DWV population diversity following *Varroa* clearance and the consequent marked reduction in DWV levels (Figure 1B), samples were selected for next-generation sequencing (NGS) analysis. Using individual bees, DWV amplicons from May (time point (TP) 1–including a pool of *Varroa* for each colony collected 24 h after treatment), June (TP2), and September (TP5) of 2019 were prepared and sequenced, with the resulting Illumina reads analysed using ShoRAH (short read alignment to haplotype) [34]. ShoRAH analyses Illumina reads in iterative windows along a specified region of the genome and clusters them into probable haplotypes, which are assigned as a percentage of the whole population. Neighbour-joining phylogenetic analysis of DWV sequence haplotypes generated by ShoRAH were performed using sequences spanning the RdRp, the helicase, and the protease (Figure S1), chosen for being known regions of recombination or sequence diversity [6,23,24,39].

**Figure 2:**
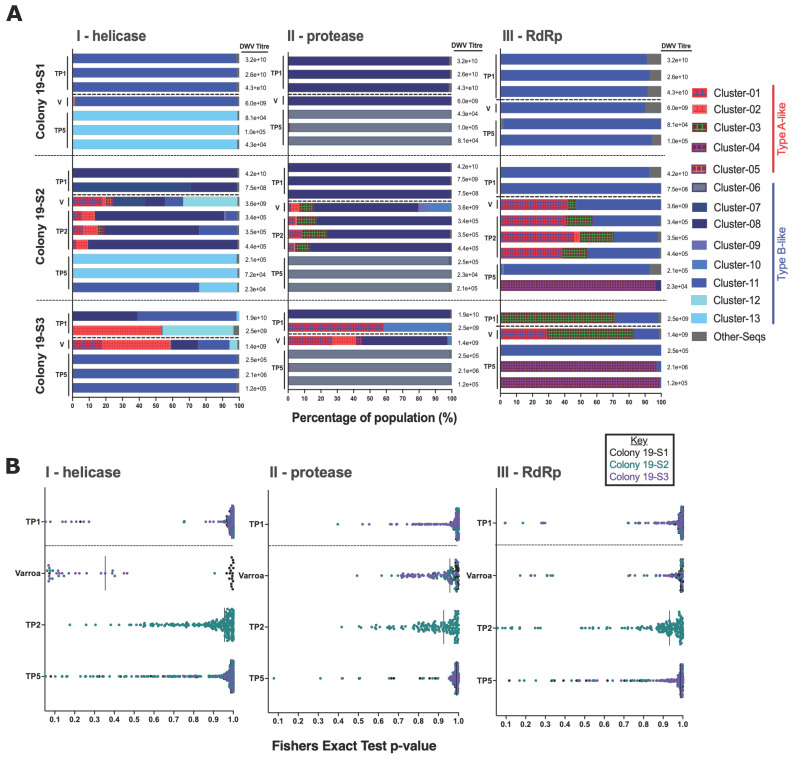
Analysis of DWV sequence diversity in shook swarm samples using ShoRAH compiled haplotypes and Single nucleotide variants (SNVs) analysis. Panel (**A**)-The different colours at each time point represent the Type A and B haplotypes from each colony, with red representing Type A variants and blue representing Type B. The Type A haplotype clusters are also cross-hatched to further distinguish them from one another. *Varroa* pools (V) are shown separately from the TP1 honeybee samples and were collected 24 h after miticide application. Haplotypes were generated from next generation sequence data and assigned to phylogenetic clusters for the helicase (I), protease (II), and RdRp (III). The bars are coloured red if they best-matched Type A-like sequences and blue if they best-matched Type B-like sequences in Figure S2. The quantified viral load in each honeybee is shown beside each bar. The dashed line indicates when the shook swarm was carried out. Panel (**B**)—SNVs from ShoRAH analysis for all samples from the helicase (I), protease (II) and RdRp (III) were analysed if present in three iterations of the modelling. Samples are coloured by colony. SNV *p*-values close to 1.0 represent a nucleotide variant present in most sequences in that dataset differing from the reference sequence. Samples with SNVs with lower *p*-value scores therefore have a greater amount of sequence variation within the sample. The vertical bar on each plot indicates the median *p*-value score for all samples. Anything with a *p*-value < 0.05 was excluded based on the defined threshold for error.

The haplotypes were assigned to ‘clusters’ based on the branches they formed during the phylogenetic analysis. These clusters were subsequently compiled as bar charts showing the percentage composition of each variant to visualise changes in diversity over time (Figure 2A). Colony 19-S1 contains only Type B sequences, and each individual contains a single viral variant, including the pooled *Varroa* sample. In colony 19-S2, the virus population at TP1 is also Type B only, but the pooled mite samples for 19-S2 and 19-S3 contained mixed populations of A and B variants. The TP2 workers for 19-S2 contained a mix of Type A and B variants. However, the population was still predominantly (>80%) Type B. Different regions of the genome aligned with different variants in colony 19-S2 and 19-S3, with the RdRp containing Type A variants, but other regions containing only Type B, suggesting viral recombinants are potentially present. By TP5, very little diversity was observed at an individual level in the samples from all three colonies.

Probabilistic clustering of the sequences combined with statistical tests for strand bias for each time point were carried out using the ShoRAH single nucleotide variant (SNV) caller [35]. This method verified that the SNVs determining sequence diversity were being called with high accuracy and were not false positives due to low sequence coverage or systematic errors during variant calling. The *p*-values of each SNV were used to generate plots for each time point (Figure 2B). The largest change in diversity was observed in colony 19-S2, as seen in the bar-plot in Figure 2A. The virus diversity in mites was similar to that in the sampled worker bees from the TP1 for all colonies. Colony 19-S1, indicated by the black dots, showed much higher SNV conservation across all time points, indicating lower virus diversity, than colonies 19-S2 and S3, but still showed an increase in diversity by TP5.

### 3.3. Changes in the DWV Sequence Diversity following Varroa Infestation of Varroa-Naïve Colonies

We installed colonies with very low *Varroa* levels in an apiary already containing highly infested colonies, in which the mite population was unmanaged, to determine what changes occur in the virus population of *Varroa*-naïve colonies post-*Varroa* infestation. Prior to the movement, the introduced ‘naïve’ colonies were treated with a miticide, and DWV levels were quantified. Only colonies with consistently low DWV (<10^6^ GE/bee) by qPCR analysis, no symptomatic workers, and very low mite levels (<10 total seven days post-treatment) were used as test colonies in this part of the study.

In two seasons, 2017 and 2019, colonies were placed within 10 metres of 15–18 mite infested colonies and the brood was sampled every 21–28 days. All changes in colony health were measured through changes in DWV level and sequence diversity. Additionally, qPCR was used to quantify the DWV in the emerging brood from each of the test colonies and from one infested neighbouring (IN) colony at the end of each season.

**Figure 3:**
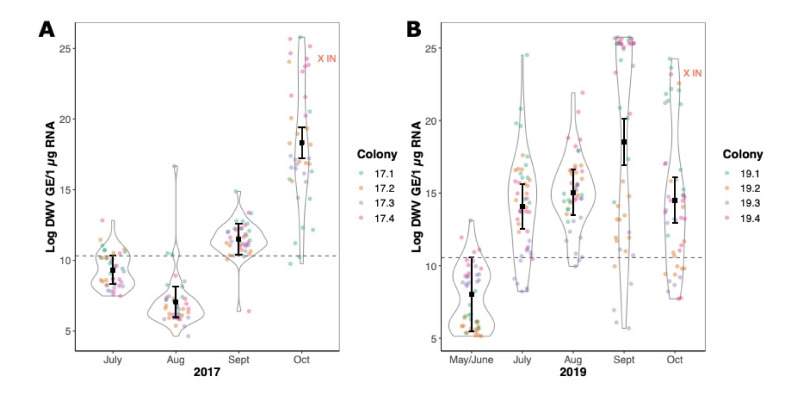
A violin plot showing the log DWV levels across multiple colonies and multiple seasons when colonies with low DWV and low Varroa levels were placed in a highly mite-infested apiary without treatment in 2017 (**A**) and 2019 (**B**). Only the emerging broods were sampled from the colonies, to ensure the bees sampled had not drifted from neighbouring colonies, so on occasions where no brood was available, samples were not taken or omitted from this analysis. Coloured circles represent individual honeybee samples from each colony (*n* = 12). The black square represents the predicted mean from the mixed-effects model for each time point with corresponding 95% confidence intervals around the mean. Estimates and 95% confidence intervals above the dashed line indicate they are above the upper 95% confidence interval from the pre-treatment timepoint. Red crosses represent the mean log DWV from an infested neighbouring (IN) colony in the apiary, which was sampled in the same manner as the naïve colonies at the end of each season (2017 *n* = 10; 2019 *n* = 12).

The DWV levels in 2017 and 2019 showed a broadly similar pattern of initially low viral loads in the first month of sampling, followed by an increase in virus levels at the subsequent sampling time points after the colonies were co-located in the mite-infested apiary. Average titres of ~10^4^ GE/μg RNA in July increased 100,000-fold to 5 × 10^9^ GE/μg RNA by October (Figure 3A). 

Both the simple linear models and the mixed models indicated that time point was a significant predictor of log DWV in sampled bees in both 2017 and 2019 (Tables S3 and S4), with a slightly different pattern in each year. In 2017, the log levels of DWV initially dropped before rising above the pre-movement sampling in the final two time points (Figure 3A). In 2019, the increase in log DWV after moving colonies into the *Varroa*-infested apiary was immediate and sustained, with all subsequent samplings containing a greater quantity of DWV (Figure 3B). The mixed effect models provided the lowest AIC in both 2017 (lm AIC = 758; GLMM AIC = 756) and 2019 (lm AIC = 1330; GLMM AIC = 1280), indicating that colonies responded differently to the *Varroa* pressure in both years. The proportion of residual variation explained by the inclusion of the random effect for the colony varied from 20% in 2017 to 38% in 2019 (Tables S3 and S4). The amount of DWV in the infested neighbouring colony (the IN red points in Figure 3A,B) broadly reflected the average observed in the test colonies at the end of each season. Furthermore, all the 2017 colonies had died within 14 months of co-location with mite-infested colonies (data not shown).

### 3.4. Analysis of DWV Diversity Changes in Newly Varroa-Infested Colonies

**Figure 4:**
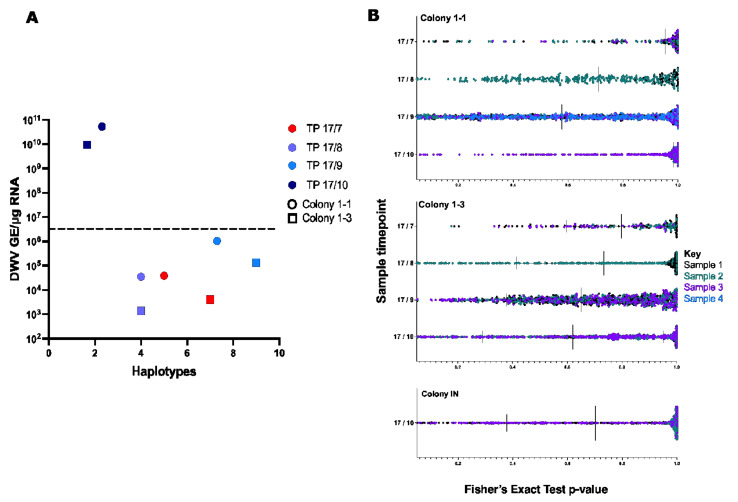
Deformed Wing Virus sequence diversity changes in two Varroa-naïve colonies. (**A**). The average DWV load from each time point and the number of haplotypes shared amongst the samples sequenced in Colony 1-1 and Colony 1-3 are compared. Samples are coloured by timepoint and shaped by colony. Dashed line indicates known threshold for symptomatic infections in developing bees [7]. (**B**). SNVs from ShoRAH analysis for all samples from colonies 1-1, 1-3 and IN were called if present in 3 iterations of the modelling, as described in Figure 2B. The different colours at each time point represent the SNVs for each honeybee sample sequenced and match the corresponding colours to the left of Figure S3. Vertical black lines show the median (larger lines) and interquartile range for the *p*-value scores.

**Figure 5:**
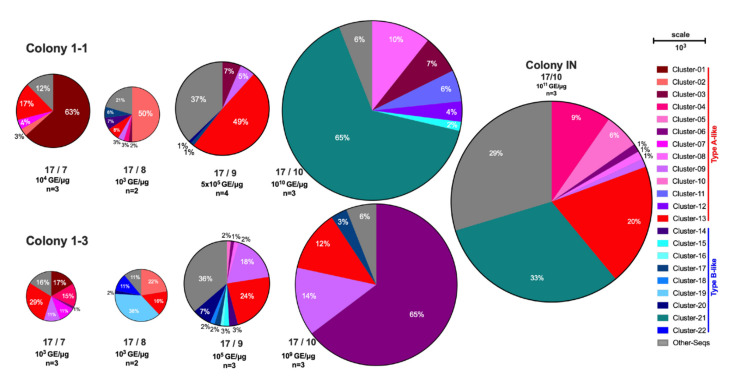
Visualisation of haplotype diversity and scale of virus level for each time point for Colony 1-1 and 1-3. Each pie chart represents a single time point for one colony, with all haplotype variants from multiple bees sequenced individually shown. The assigned clusters were coloured shades of red if they best matched Type A reference genomes and blue if they best matched Type B reference genomes. The size of the pie is determined by the DWV genome equivalents, shown below each pie, scaled using the circle diameter. The percentage that each haplotype makes up is shown in each segment. The segments reflect the granularity determined by ShoRAH and it is possible that other variants not detected by ShoRAH are present at low levels. The number of individual samples collated for each time point are shown below the virus titre as *n* = x.

To investigate whether the sequence diversity of DWV changed as mites infested the *Varroa*-naïve colonies in this study, selected amplicons from individual brood samples from two colonies, 1-1 and 1-3, and an infested neighbouring (IN) colony were sequenced from the four time points in year one (2017). Haplotypes were assigned to clusters, as described above, and compiled into bar plots to visualise changes over time (Figure S3). Typically, as the DWV yield increased to >5 × 10^6^ GE/μg RNA, the number of haplotypes decreased (in 4/6 samples by TP3 and on average—Figure S2). The dominant variant was colony specific by the end of the season, with a Type A variant observed in colony 1-1 and a Type B variant in colony 1-3. A Type A variant had been present in both colonies initially but was not observed at later time points. In both colonies, the number of viral haplotypes decreases significantly when the virus titre increases >5 × 10^6^ GE/μg RNA (Figure 4A). To better visualise the changes in virus titre and haplotype diversity, they were plotted, with the radius scaled to indicate the viral load (Figure 5). There is a single dominant variant at the colony level, but diversity remains within the virus population.

The samples from the mite-infested co-located colony showed high diversity in 2/3 samples, despite having high DWV loads (>10^8^ GE/μg RNA) (Figure S3). The one clonal sample from the infested colony aligned with Type B variants, like those present in colony 1-3. Despite being a colony with a longer history of mite infestation, there was a similar level of heterogeneity between the samples sequenced.

As per Section 3.2, probabilistic clustering was performed, combined with statistical tests for strand bias for each time point, using the ShoRAH single nucleotide variant (SNV) caller. The SNV analysis (Figure 4B) indicated that TP0 samples had relatively low sequence diversity, but that diversity increased at TP1 and TP2 before decreasing again, coinciding with the virus titres significantly increasing at TP3.

Additionally, Sanger sequencing was used to examine the virus population in the year one samples (2017) (Figure S4A). The dominant variant in each sample, as identified by Sanger sequencing, broadly correlated with the NGS data, indicating that a more economical analysis method could be used to determine the predominant virus in the population if the underlying virus diversity was not of interest. Sanger sequencing was then used to evaluate any change in the dominant variant in the year two samples (2019) (Figure S4B), with a similar colony-specific variant dominance but with a greater number of sequences for Type B-like variants observed.

## 4. Discussion

The majority of overwintering honeybee (*Apis mellifera*) colony losses, which can exceed 30% of all managed colonies [11,40], are attributed to infestations of *Varroa* destructor and the consequent transmission of Deformed wing virus (DWV). *Varroa* feeding results in direct systemic transmission of DWV, an endemic virus that is usually spread horizontally in the colony during larval feeding and trophallaxis [3,4] and vertically by queen infection [41]. Potentially because of bypassing normal immune protective mechanisms in the gut, the *Varroa*-transmitted virus replicates to significantly elevated levels, causing overt disease in recipient pupae, and may exhibit a marked reduction in DWV population diversity [5,6,7].

Beekeeping in the UK is unregulated, and consequently, colony management techniques and *Varroa* treatment choices vary markedly between beekeepers. The approved miticides are not 100% effective, meaning good colony management and regular *Varroa* monitoring are required to prevent losses caused by uncontrolled mite replication. Additionally, phoretic mites are acquired through the movement of bees between colonies, for example, drifting and robbing workers, resulting in re-infestation of colonies in which mite levels had previously been controlled [12,13]. To combat this, landscape-scale mite control is recommended, but the reality is that neighbouring beekeepers generally treat colonies at different times and with different treatments or may omit treatment altogether, leading to conditions in which environmental transmission of *Varroa* occurs. Additionally, some beekeepers move their colonies as part of commercial beekeeping practices or for seasonal pollination, such as ‘oilseed rape’ (canola) or heather.

In this study we demonstrated that *Varroa*-naïve colonies, when co-located with mite-infested colonies, rapidly exhibit elevated DWV levels and changes in their virus population as a consequence of acquiring mites from nearby colonies. In two repeated seasons, the introduced colonies either died within 14 months or exhibited the very high virus levels associated with symptomatic disease and overwintering colony losses (Figure 3). Previous studies have reported increases in DWV titre and a reduction in virus population diversity at the colony level in association with *Varroa* infestation [5]. However, changes in the virus population of individual workers in the colony has not been examined, and we were keen to investigate this to help elucidate if and why certain variants eventually dominate the population. 

Prior to re-location to the *Varroa*-infested apiary, the *Varroa*-naïve colonies contained low levels (~10^3^–10^4^ GE/μg RNA) and mixed populations of Type A and B variants of DWV (Figure 4 and Figure S4). Three months after installation in the *Varroa*-infested apiary, all samples tested contained markedly elevated viral loads (>10^8^ GE/μg RNA). In addition, two-thirds of the workers analysed contained a single dominant DWV variant that had not exceeded the 3% detection threshold in the initial colonies (Figure 4). Two of the six workers sampled in this study exhibited a mixed virus population three months post-co-location. However, the make-up of this population differed significantly from that at the start. Recent studies have shown that dominance of a single DWV type or strain is not the invariant consequence of *Varroa* infestation [19,20], suggesting that there may be stochastic events that influence the changes observed in the virus population following mite infestation.

Virus population changes were not consistent between colonies in the workers sequenced three months after colony movement to the *Varroa*-infested apiary. In each colony, 2/3 workers contained a similar virus haplotype. However, this differed between colonies (one being Type A and the other being a Type B virus), and they differed in the titre of this dominant virus, which varied from ~10^7^-10^11^ GE/μg RNA (Figure 4). The remaining workers sampled at the same time points (1/3 in each colony) contained mixed virus populations, though of a similar titre. The dominance of both Types A and B in individual workers, rather than total replacement of one type with another, supports the observed similarities in infectivity of Type A and B during in vitro injection studies [7,25,39]. Together with the earlier timepoint samples from the same colonies, the results suggest that the cumulative virus population in the colony reflects a significant level of variation in individual workers and that their history of virus exposure (through parasitism by *Varroa*) may result in markedly different outcomes. However, the dominance of cluster-21 by the final time points in colony 1-1 is similar to one sample from the infested neighbouring colony data (Figure 4 and Figure 5), suggesting robbing or drifting from the IN colony may have distributed the mites/bees to colony 1-1 and facilitated the transmission of this variant. 

The SNV analysis (Figure 4B) indicated the virus populations of the two colonies went through an initial expansion of virus diversity, presumably as mites infested the colonies and introduced new variants before a subsequent diversity bottleneck occurred as the virus levels increased. Then, the diversity in the majority of the samples decreased again as some viruses became dominant due to their particularly transmissibility or transmission history [21]. The experimental design means we could not discriminate between repeated multiple mite transmission events, each introducing a limited range of new viruses and a limited incursion of mites carrying a wide diversity of viruses. The apiary layout, ~18 colonies within 20 m^2^, and sampling intervals make the first of these two possibilities more likely. The *Varroa*-infested colonies in the apiary are maintained with minimal intervention and no miticide treatment (with consequent high colony losses in some years).

Unmanaged mite populations can rapidly expand and threaten colony health. Mite daily reproductive rates are reported as 0.017–0.021, and the population doubling time has been estimated at ~33–41 days, meaning a population of ~100 mites in spring could reach >3000 mites by autumn assuming uninterrupted brood rearing [42]. To combat the damaging consequences of mite infestation, beekeepers must monitor mite levels and intervene where necessary. Since the majority of approved miticides are only effective against phoretic mites, whereas the majority of mites are resident in capped cells, there is a need for prolonged miticide treatment. We reasoned that the combination of a ‘shook swarm,’ a standard beekeeping management technique that separates all adult bees from the (potentially *Varroa*-infested) brood, coupled with miticide application would achieve the maximum impact on the mite population and enable us to investigate the post-treatment consequences for the virus population. The latter is an important consideration; if virus levels remain high, colony survival may still be threatened, as observed by Highfield et al., 2009, where some colonies with low mites had high DWV and died over winter. Furthermore, such colonies may be particularly susceptible to damaging levels of virus transmission should they become re-infested with *Varroa* following environmental exposure, as observed by [18].

For operational reasons, we conducted the shook swarm and simultaneously treated colonies with Apivar, which contains the synthetic miticide amitraz. *Varroa* levels counted on the trays underneath the colonies declined rapidly to <100 mites total within five days and <10 mites total within nine days (Figure 1A). In the absence of any further intervention, natural mite drops, a consequence of allogrooming in the colony, was very low for the remainder of the season. These results were not unexpected and reflected the efficacy of amitraz-containing miticides and the relative isolation of the treated colonies in our research apiary. It seems likely that a single-shot or short-duration miticide like trickled/vaporised oxalic acid or formic acid would be an equally appropriate treatment for a mite-infested shook swarm.

Importantly, in addition to the rapid drop in mite numbers, the virus levels also significantly reduced when tested one month after conducting the combined shook swarm and miticide application and remained low for the entire season. Pre-treatment virus levels averaged 8 × 10^7^ and 5 × 10^9^ GE/μg RNA in the two years and were reduced to 4 × 10^3^ and 6 × 10^4^ GE/μg RNA in the first month post-treatment, respectively (Figure 1B). The viral loads post-treatment remained consistently below a level of 5 × 10^6^ GE/μg RNA, a threshold we have previously associated with symptomatic infection [7], for the rest of the season during which brood rearing continued.

Recent NGS analyses of DWV infections have shown individual bees carrying high viral loads can contain either near-clonal or diverse virus populations [19,20]. Using ShoRAH analysis of pre- and post-shook swarm emerging worker samples, we examined whether the removal of *Varroa* and consequent reduction of viral load in the colony also modified the virus population. Pre-treatment, individual newly emerged workers contained near-clonal virus populations, but the dominant variant differed between the colonies (Figure 2). The mites, sampled as a pool and thereby likely explaining the greater range of viruses present, carried a mixed population of Types A and B DWV in two colonies (19-S2 and S3). These variants were detected in the first brood samples examined post-treatment, indicating that both Type A and B had been passed from mite to adult worker to brood without an apparent selective advantage. This supports findings of Type B-like variant replication in mites [7] and non-propagative transmission of Type A by mites [28], allowing both variants to survive in the honeybee population. 

Five months post-treatment, the virus population had changed in colonies 19-S2 and S3, and the workers all contained a dominant variant (>95%). Like the infested colonies (Figure 4), this suggests the colonies went through a bottleneck event where diversity was lost. The dominant variants varied between colonies and from worker to worker within colonies, indicating that diversity changes may have occurred through a stochastic process with no selective advantage for a particular variant. Distinct variants, as defined by the haplotype assignment from ShoRAH, can be classified based on a handful of SNPs. These changes do not necessarily confer a phenotypic difference between variants.

As not all the mid-season brood were sequenced, when this virus population shift took place is unclear. However, it likely occurred during feeding/trophallaxis, where a high threshold for virus transmission to larvae meant only a subset of a low-level DWV population was transmitted. In turn, variants were lost from generation to generation. This high transmission threshold supports the findings that the highly infected adults did not transmit high virus numbers to the first brood samples post-treatment. The sample size in both experiments was relatively small and may only represent a snapshot of the true virus population changes in these colonies. However, these changes could still be reflective of stochastic changes occurring at the colony level.

Recent reports, based on field study sample analysis, have suggested that DWV Type B may be increasing in prevalence and, in some cases, superseding Type A variants. Based on the sequencing analysis from the infested colonies (Figure 4 and Figure S4), the proportion of Type B sequences increased between the two years (2017 and 2019). Additionally, the sequence analysis of the shook swarm data (Figure 2), also taken in 2019, was predominantly composed of Type B variants, supporting the findings of its increased prevalence from England and the USA [27,28,43].

The results of this study clearly demonstrate the rapid impact of *Varroa* infestation on the DWV load and population at an individual and colony level when they are left untreated and the consequences of this on the colony’s health. Analysis of viruses following the placing of *Varroa*-naïve colonies in a mite-infested environment shows the dramatic influence infesting mites have on both the level and the composition of the virus population. This is further emphasised when we investigated what happens to the virus population after the targeted removal of *Varroa* by a colony management intervention that should remove up to 90% of *Varroa* coupled with application of an approved miticide. This combination effectively and rapidly reduced *Varroa* and DWV levels to those associated with a ‘healthy’ colony within a single brood cycle, and no symptoms or large increases in virus levels were observed for the rest of the study. These experiments, while providing insights into dynamic virus population changes, further emphasize the importance of good beekeeping practices and mite management techniques to improve and maintain honeybee colony health.

## Data Availability

All of the NGS data files used in this paper are publicly available in the SRA (short read archive) of NCBI, which is accessible under BioProject ID: PRJNA811438.

## Supplementary Materials

The following supporting information can be downloaded at: https://www.mdpi.com/article/10.3390/v14081597/s1, Figure S1: Neighbour joining phylogenetic analysis of ShoRAH haplotypes from 2019 Shook Swarm colonies. Figure S2: Relative viral loads of colonies left in a highly infested apiary over time. Figure S3: Analysis of DWV sequence diversity in Varroa-naive samples using ShoRAH compiled haplotypes. Figure S4: Phylogenetic analysis of diversity changes over time in *Varroa*-naïve colonies. Table S1: Summary of simple linear model to investigate DWV changes after shook swarm application. Table S2: As S1 for the mixed effects model. Table S3: Summary of simple linear model to investigate DWV changes after moving low mite colonies into a high mite apiary. Table S4: As S3 for the mixed effects model.
