# Supplementary material for: Quantitative and Qualitative Changes in the Deformed Wing Virus Population in Honey Bees Associated with the Introduction or Removal of Varroa destructor
Source: Viruses. 2022 Jul 22;14(8):1597. doi: 10.3390/v14081597 (PMC9332399; doi:10.3390/v14081597)
Supplement: Supplementary file 1 [file viruses-14-01597-s001.zip › viruses-1682782-supplementary.pdf]

Supplementary Files - Quantitative and qualitative changes in the Deformed wing virus population in honey bees associated with the introduction or removal of *Varroa destructor.*

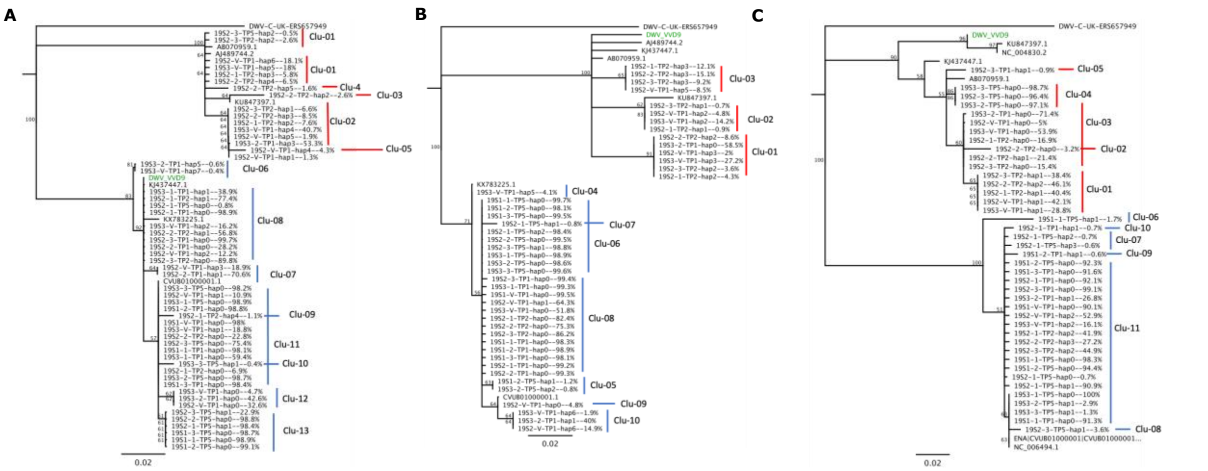

Figure S1: Neighbour joining phylogenetic analysis of ShoRAH haplotypes from 2019 Shook Swarm colonies. *The (**A**) helicase (**B**) protease and (**C**) RdRp were analysed. Samples are labelled as 19Sx-y-TPz-hap% where x = colony number, y = sample number and z = time point number. Sample numbers labelled 'V' are from* Varroa*. The hapx_xx% indicates the haplotype number from ShoRAH and the percentage of the population the sample corresponds to. The blue and red bars indicate the Type B and Type A sequence cluster assignment used to make the bar plots in Figure 2. Reference genomes are shown by their NCBI accession number. The green sequence (DWV_VVD9) was used for the haplotype alignment. The genetic distance model for the phylogenetic analysis was Tamura-nei and no outgroup was included. The phylogeny were generated from 1000 bootstrap iterations and the trees were generated using majority greedy clustering. DWV Type C (ERS657979) was used as the outgroup in each analysis. Sequences were trimmed and aligned and phylogenetic analysis carried out using Geneious Prime 2019.1.*

	Timepoint		2017 Colonies				2019 Colonies			
			1-1	1-2	1-3	1-4	2-1	2-2	2-3	2-4
2017	July	17/7	3.7E+04	5.1E+03	3.7E+03	3.2E+03				
	August	17/8	8.2E+03	7.5E+02	1.7E+06	1.4E+03				
	September	17/9	5.4E+05	6.3E+04	8.0E+04	1.7E+05				
	October	17/10	1.6E+10	2.9E+09	3.0E+07	7.93E+06				
2018	May/June	18/6			1.4E+07	2.6E+03				
	July	18/7			2.2E+10	2.3E+03				
	August	18/8				1.6E+05				
	September	18/9				1.5E+09				
2019	May	19/5					8.81E+02	3.00E+02	5.01E+04	4.49E+04
	July	19/7					3.86E+09	1.01E+07	3.89E+04	2.00E+06
	August	19/8					7.70E+06	1.04E+07	5.46E+06	3.83E+08
	September	19/9					7.02E+10	1.23E+06	8.76E+09	9.33E+10

Key
healthy (10-10^6)
At risk (10^6-10^8)
High risk (>10^8)
deceased

Figure S2 - Relative viral loads of colonies left in a highly infested apiary over time. *Colour scale represents increasing viral load from yellow to red. Black bars represent points where colonies have died.* Average quantified viral load shown for each colony at each time point.

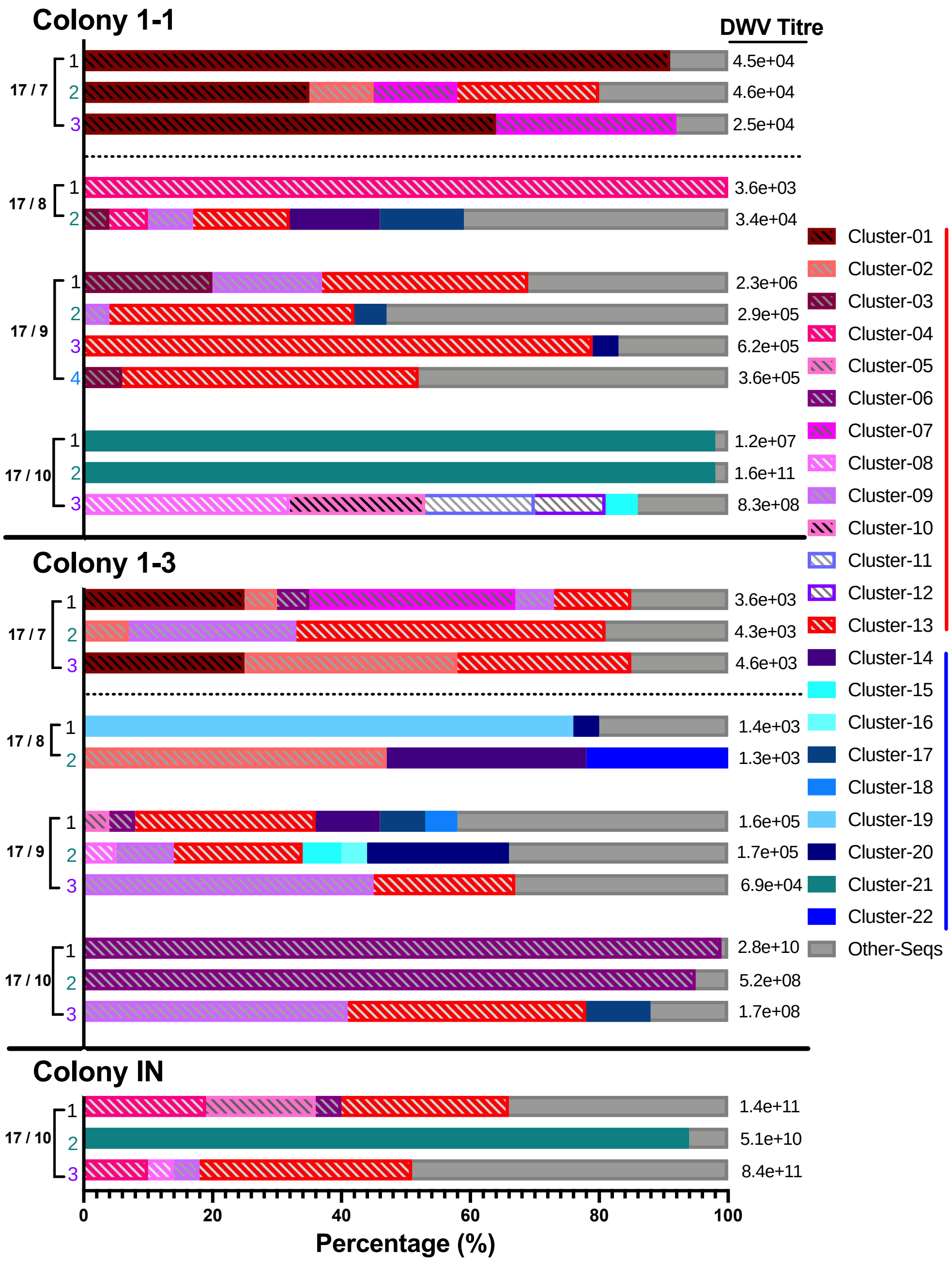

***Figure S3* – Analysis of DWV sequence diversity in Varroa-naive samples using ShoRAH compiled haplotypes.** The clusters were compiled in to barplots to visualize changes in diversity over time for colony 1-1, 1-3 and a neighbouring infested colony (IN). The assigned clusters were coloured shades of red if they best matched Type A reference genomes and blue if they best matched Type B reference genomes. All Type A clusters are cross-hatched. 'Other_Seqs' are ShoRAH haplotypes below the 3% error threshold determined using a spiked control (data not shown). The quantified viral load of each honey bee is shown beside each bar. The dotted line indicates when the colonies were moved to the infested site.

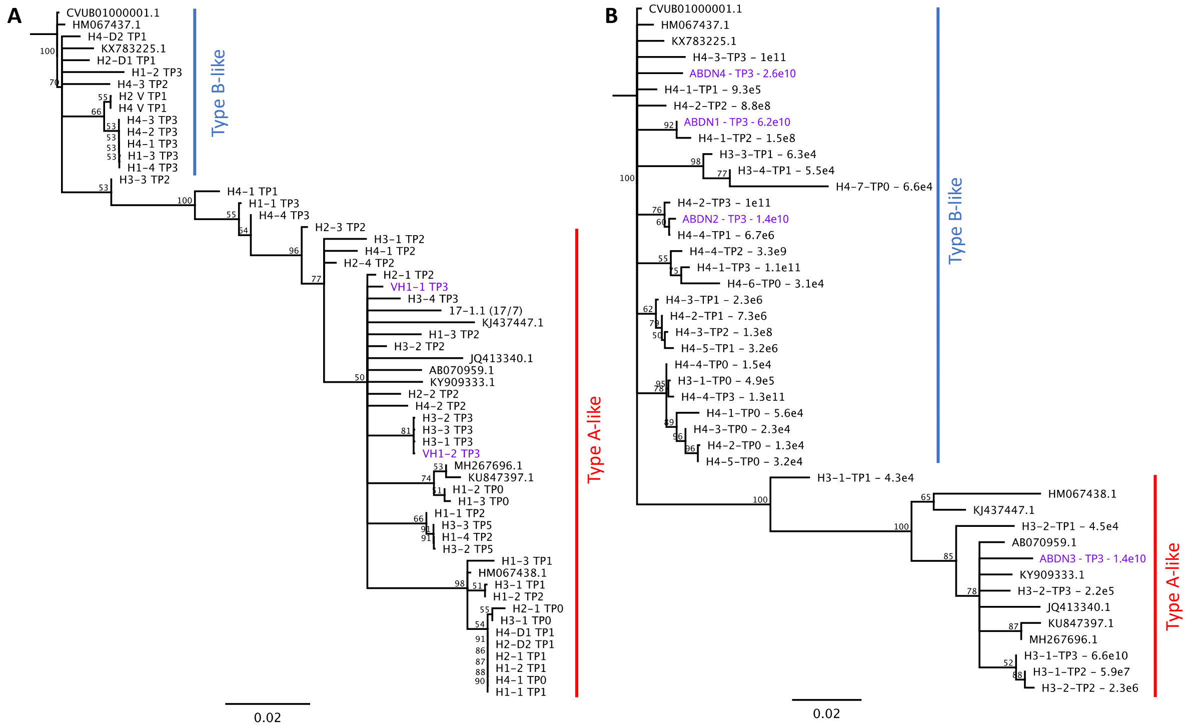

Figure S4 – Neighbour joining phylogenetic analysis of diversity changes over time in Varroa-naïve colonies. *Sanger sequences of helicase region (4500-5500bp) from Year 1 (A) & Year 2 (B) colonies. Samples are named by colony (Hx), sample number and time point (TPx). Samples from the neighbouring infested colony are shown in purple for each year. Reference genomes are shown by their accession number. The genetic distance model for the phylogenetic analysis was Tamura-nei and the outgroup was CVUB01000001.1. The phylogeny were generated from 1000 bootstrap iterations and the trees were generated using majority greedy clustering. Sequences were trimmed and aligned and phylogenetic analysis carried out using Geneious Prime 2019.1.*

Table S1 – *Summary of linear model outputs for 2018 and 2019 assessing the change in log DWV titre from the brood samples taken before the shook swarm treatments (May), and subsequent samples.*

Year	R^2 (adj)	F value	Fixed effect month	T value	P value
**2018**	0.88	316.5	May	79.7	<0.001
			June	-33.34	<0.001
			July	-29.66	<0.001
			August	-32.57	<0.001
			September	-28.52	<0.001
			October	-27.37	<0.001
**2019**	0.62	63.44	May	39.18	<0.001
			June	-12.95	<0.001
			July	-13.77	<0.001
			August	-11.12	<0.001
			September	-11.66	<0.001
			October	-15.50	<0.001

Table S2 – *Summary of mixed effect model outputs for 2018 and 2019 assessing the change in log DWV titre from the brood samples taken before the shook swarm treatments (May), and subsequent samples.*

Year	Random effect colony (St Dev / Residual)	Fixed effect month	T value	P value
**2018**	0.0006 / 1.189	May	79.66	<0.001
		June	-33.34	<0.001
		July	-29.66	<0.001
		August	-32.57	<0.001
		September	-28.52	<0.001
		October	-27.37	<0.001
**2019**	0.916 / 2.565	May	25.88	<0.001
		June	-13.48	<0.001
		July	-14.28	<0.001
		August	-11.59	<0.001
		September	-12.09	<0.001
		October	-16.27	<0.001

Table S3 – *Summary of linear model outputs for 2017 and 2019 assessing the change in log DWV titre from the brood samples taken before colonies were moved to a mite infested apiary (July 2017 and May 2019), and subsequent monthly samples.*

**Year**	**R^2 (adj)**	**F value**	**Fixed effect month**	**T value**	**P value**
**2017**	0.73	147.4	July	23.24	<0.001
			August	-3.96	<0.001
			September	3.83	<0.001
			October	15.90	<0.001
**2019**	0.39	36.9	May/June	12.10	<0.001
			July	6.98	<0.001
			August	8.04	<0.001
			September	11.90	<0.001
			October	7.44	<0.001

Table S4 – *Summary of mixed effect model outputs for 2017 and 2019 assessing the change in log DWV titre from the brood samples taken before colonies were moved to a mite infested apiary (July 2017 and May 2019), and subsequent monthly samples.*

**Year**	**Random effect colony (St Dev / Residual)**	**Fixed effect month**	**T value**	**P value**
**2017**	0.636 / 2.474	July	18.49	<0.001
		August	-4.06	<0.001
		September	3.93	<0.001
		October	16.30	<0.001
**2019**	2.32 / 3.78	May/June	6.18	<0.001
		July	7.70	<0.001
		August	8.86	<0.001
		September	13.03	<0.001
		October	8.18	<0.001
